# Supplementary material for: Identification of Freezing-Responsive microRNAs and Their Targets in Chinese Jujube by Small RNA and Degradome Sequencing
Source: Int J Mol Sci. 2026 Sep 4;27(17):7912. doi: 10.3390/ijms27177912 (PMC13566890; doi:10.3390/ijms27177912)
Supplement: Supplementary file 1 [file ijms-27-07912-s001.zip › Supplementary Material S2/novel_miR_20.pdf]

Provisional ID	:	novel_miR_20
Score total	:	624.6
Score for star read(s)	:	3.9
Score for read counts	:	615.5
Score for mfe	:	3.6
Score for randfold	:	1.6
Score for cons. seed	:	
Total read count	:	1219
Mature read count	:	396
Loop read count	:	820
Star read count	:	3

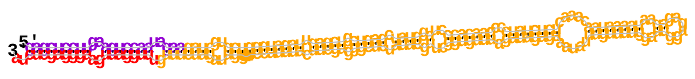

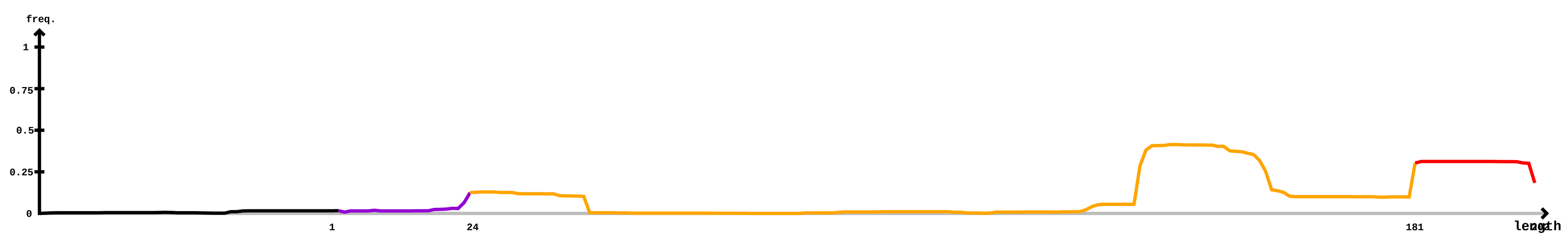

Star — Mature

```
5'- cgagagagagagagagagagagagagagaucuaaaaaauuaggguuagaaguggagcuccuugaaguccaauagaagaucgugcuggguagauugagcugcugagcuauggaucccacaagcccuaucucucaaacaauaaaaauaagagagagguuagguuuuguuuauaaagagauagauuuguggcuugcauaucucgggagcuucauuuaccuauagaguuagaucuuuguuuggauugaagggagcucuac -3'   obs
    cgagagagagagagagagagagagagagaucuaaaaaauuaggguuagaaguggagcuccuugaaguccaauagaagaucgugcuggguagauugagcugcugagcuauggaucccacaagcccuaucucucaaacaauaaaaauaagagagagguuagguuuuguuuauaaagagauagauuuguggcuugcauaucucgggagcuucauuuaccuauagaguuagaucuuuguuuggauugaagggagcucuac          exp
    .......................(((((........)))))....((((((((((((..(((((((..(((((((..((((((((((.(((((.(((((.((((.(..(((((((..((((((((.....((((((((..((......))..))))))))).....))))))))).)))))))..).)))).))))).))))).))))))))....)))..)))))))..)))))))..)))))))))))   reads   mm   sample
```

alignment	reads	mm	sample
cgagagagCgagagagagagag...	1	1	JCK-2
cgUgagagagagagagagaga...	2	1	LT-1
Ggagagagagagagagagagagagagau...	1	1	JCK-1
.gagagagagagNgagagagagaga...	1	1	JCK-1
.........gagagUgagagagagagauc...	1	1	LCK-3
......................agagagaucuaaaaaauuaggguuagaagug...	1	0	LCK-3
.......................gaAagaucuaaaaaauuaggguuagaagug...	1	1	LT-3
...................................aaauuaggguuagaagugg...	11	0	JT-1
.....................................auuaggguuagaaguggagcucc...	2	0	JCK-3
.....................................auuaggguuagaaguggagcucc...	3	0	JCK-2
.....................................auuaggguuagaaguggagcucc...	1	0	JCK-1
......................................uuaggguuagaaguggagcuc...	1	0	JCK-2
....gagcuccuugaaguNcaaua...	1	1	LCK-1
....gaNcuccuugaaguccaauag...	1	1	LT-2
....gagcuccuugaaguccaauagUa...	1	1	LT-1
....gcuccuugaaguccaauagaagaucgug...	6	0	LCK-2
....gcuccuugaaguccaauagaagaucgug...	3	0	LCK-1
....cuugaaguccaauagaagauc...	1	0	LT-3
....cuugaaguccaauagaagauc...	5	0	JCK-1
....uugaaguccaauagaagaucgugc...	1	0	LT-1
....uccaauagaagaucgugcuggg...	1	0	LT-3
....aauagaagaucgugcugggua...	2	0	LT-3
....aauagaagaucgugcugggua...	6	0	LCK-3
....aauagaagaucgugcugggua...	1	0	JCK-2
....aauagaagaucgugcuggguagau...	1	0	LT-2
....auagaagaucgugcuggguag...	1	0	LT-3
....uagaagaucgugcuggguaga...	1	0	LT-3
....uagaagaucgugcuggguagauu...	1	0	JCK-2
....agaagaucgugcugggua...	5	0	LCK-3
....agaagaucgugcuggguagauugagcug...	1	0	LCK-3
....aagaucgugcuggguagauu...	1	0	LT-3
....aagaucgugcuggguagauug...	2	0	JCK-2
....aagCucgugcuggguagauug...	1	1	LT-2

Star **Mature**

cgagagagagagagagagagagagagaucuaaaaauuaggguuagaagug**gagcuccuugaaguccaauagaa**gaucgugcuggguagauugagcugcugagcuauggaucccacaagcccuaucucucaaacaauaaaauaagagagagguuagguuuuguuuauaaagagauagauuuguggcuugcauaucucgggagcuucauuuaccuauagaguuagaucuuug**uuuggauugaagggagcucua**c

aagaucgugcuggguagauug	3	0	JCK-3
aagaucgugcuggguagauug	18	0	LT-2
Cagaucgugcuggguagauug	1	1	JCK-2
aagaucgugcuggguagauug	8	0	LT-1
aagaucgugcuggguagauug	12	0	LT-3
agaucgugcuggguagauug	30	0	LCK-3
agaucgugcuggguagauug	17	0	LT-2
agaucgugcuggguagauug	1	0	LT-1
agaucgugcuggguagauug	6	0	LT-3
agaucgugcuggguagauug	8	0	JCK-2
agaucgugcuggguagauug	7	0	JCK-3
agaucgugcuggguagauug	8	0	JCK-1
aucgugcuggguagauug	1	0	LCK-3
aucgugcuggguagauug	2	0	LT-2
aucgugcuggguagauug	1	0	LT-1
aucgugcuggguagauug	1	0	JCK-2
aucgugcuggguagauugagcug	1	0	LT-1
gugcuggguagauugagcugcA	2	1	JCK-2
agcugcugagcNauggauccc	1	1	JT-2
ugagcuauggaucccacaagc	1	0	LT-3
caaacaauaaaauaagagagagguu	2	0	LT-3
caaacaauaaaauaagagagagguu	1	0	JCK-2
caaacaauaaaauaagagagagguu	1	0	JCK-1
aauaaaauaagagagagguuagg	1	0	LT-2
auaaaauaagagagagguuag	1	0	JCK-3
auaaaauaagagagagguuag	1	0	LT-1
auaaaauaagagagagguuag	3	0	JCK-2
uaaaauaagagagagguuagguu	1	0	LT-3
uaagagagagguuagguu	1	0	JCK-2
aagagagagguuagguuuuguuuauaaag	1	0	JCK-2
uguuuauaaagagauagauuugugg	1	0	JCK-1
uguuuauaaagagauagauuugugg	1	0	JCK-2
guuuauaaagagauagauuugugg	1	0	JCK-3
guuuauaaUgagauagauuugugg	1	1	LT-1
guuuauaaUgagauagauuugugg	3	1	LT-2
guuuauaaagagauagauuugugg	2	0	JCK-2
uaaUgagauagauuuguggcuugc	1	1	LT-1
gauagauuuguggcuugcau	2	0	LT-1
uagauuuguggcuugcaua	1	0	LT-3
gauuuguggcuugcauaucucggg	8	0	JCK-3
gauuuguggcuugcauaucucggg	1	0	LT-1
gCuuuguggcuugcauaucucggg	1	1	JCK-2
gauuuguggcuugcauaucucggg	4	0	JCK-2
gauuuguggcuugcauaucucggg	2	0	JCK-1
auuuguggcuugcauaucu	1	0	LT-1
auuuguggcuugcauaucucg	6	0	LCK-3
auuuguggcuugcauaucucg	1	0	LT-3
auuuguggcuugcauaucucgg	1	0	LT-1
auuuguggcuugcauaucucggg	6	0	JCK-3
auuuguggcuugcauaucucggg	5	0	JCK-2
auuuguggcuugcauaucucggga	3	0	JCK-1
auuuguggcuugcauaucucggga	1	0	JCK-2
uuuguggcuugcauaucucg	3	0	LT-3
uuuguggcuugcauaucucggg	6	0	JCK-2
uuuguggcuugcauaucucggg	2	0	JCK-3
uuuguggcuugcauaucucgggag	3	0	JCK-3
uuguggcuugcauaucucggU	3	1	LT-3
cuugcCuaucucgggagc	1	1	LT-3
cuNgcauaucucgggagc	2	1	LT-2
cuugcauaucucgggCgc	2	1	LT-2
cuugcauaucuAgggagc	1	1	LT-1
cuugcauaucucgggCgc	1	1	LT-1
cuugcauaucuAgggagc	2	1	LT-3
cuugAauaucucgggagc	1	1	LT-3
Uuugcauaucucgggagc	1	1	LT-1
cuugcauaucuNgggagc	1	1	JCK-1
cuugcauaucucgNgagcu	1	1	JT-2
cuugcauaucucgggCgcu	1	1	JCK-2
cuugcauaucucgggCgcu	1	1	LT-1
cuugcCuaucucgggagcu	1	1	LT-2

Star Mature

cgagagagagagagagagagagagagaucuaaaaauuaggguuagaagugggagcuccuugaaguccaauagaagaucgugcuggguagauugagcugcugagcuauggaucccacaagcccuaucucucaaacaauaaaauaagagagagguuagguuuuguuuauaaagagauagauuuguggcuugcauaucucgggagcuucauuuaccuauagaguuagaucuuuguuuggauugaagggagcucuac

cuugcauCucucgggagcu	1	1	LCK-3
cuugcauaucucgggagUu	1	1	LT-2
cuugcauaucucgggagcG	3	1	LCK-3
cuugcauaucucggNagcuu	1	1	LCK-1
cuugcCuaucucgggagcuu	1	1	JCK-2
cuugcauCucucgggagcuu	1	1	JT-1
cuugcauaucucgggagcuC	2	1	LT-3
cuugcauaucucgggagcuA	1	1	LT-1
cuugcauaucucggAagcuuc	1	1	JCK-3
cuugcauaucucgggagcuCc	1	1	JCK-1
cuugcCuaucucgggagcuuc	1	1	LT-2
cuNgcauaucucgggagcuuc	1	1	LT-2
cuugcauaucCcgggagcuuc	1	1	JCK-2
Uuugcauaucucgggagcuuc	1	1	JCK-1
cuugcCuaucucgggagcuuc	3	1	JCK-2
cuuAcauaucucgggagcuuc	1	1	JCK-1
cuugcauaucucgggagcuuU	11	1	JCK-2
cuugcauaucucgggagcCuc	1	1	LT-3
cuugcauCucucgggagcuuc	4	1	JCK-2
cuugcauaucucgggagcGuc	1	1	JCK-2
cuugcauaucucgggaAcuuc	1	1	JCK-3
cuugcauaucucgggagcuuA	3	1	LT-1
cuugcauaucucgggagcuNc	1	1	JCK-1
cuugcaCaucucgggagcuuc	1	1	JCK-1
cuugcauCucucgggagcuuc	1	1	JCK-1
cuugcauaucucgggCgcuuc	1	1	JCK-1
cCugcauaucucgggagcuuc	1	1	LT-3
cuugcauaucucgggagcuuU	9	1	JCK-1
cuugcauaucucgggagcuuU	7	1	LT-2
cuugcauaucucgggagcuuU	11	1	LT-3
cuugcauaucucggUagcuuc	1	1	LCK-3
cuugcauaucGcgggagcuuc	1	1	JCK-1
cuuNcauaucucgggagcuuc	1	1	JCK-1
cuugcauaucuAgggagcuuc	1	1	JCK-3
cNugcauaucucgggagcuuc	1	1	JT-1
cuugcauCucucgggagcuuc	1	1	LT-2
cuuAcauaucucgggagcuuc	1	1	LT-2
cuugcauaucucgggagcuuG	1	1	JCK-1
cuugcauaucucgggagcuuU	4	1	LT-1
cuugcauauAucgggagcuuc	1	1	JCK-2
cuugcauaucuAgggagcuuc	1	1	JCK-2
cuugcauaucucgggCgcuuc	1	1	LT-2
cuugcauaucucggAagcuuc	1	1	LT-1
cuGgcauaucucgggagcuuc	1	1	LT-2
cuugcauCucucgggagcuuc	1	1	JT-2
cuugcauaucucgggagcCuc	1	1	JCK-3
Uuugcauaucucgggagcuuc	2	1	LT-3
cNugcauaucucgggagcuuc	1	1	JCK-1
cuugUauaucucgggagcuuc	1	1	JCK-3
cuugcauaucucgggagcuuU	13	1	JCK-3
cCugcauaucucgggagcuuc	1	1	JCK-2
cuugcauaucuAgggagcuuc	1	1	JCK-1
cuugcauaucucgggagcuuA	1	1	JCK-3
cuugcCuaucucgggagcuuc	4	1	JCK-3
cuugcauaucuNgggagcuuc	1	1	JT-1
cuugUauaucucgggagcuuc	1	1	JCK-1
cuugcauaucucgggagcuuA	5	1	LT-2
cuugcauaucCcgggagcuuc	2	1	JCK-1
cuugcauaucucgggagcuuA	1	1	JT-1
cuugcauaCcucgggagcuuc	2	1	JCK-2
cuugcauaucucgggCgcuuc	1	1	LT-3
cuugcauCucucgggagcuuc	1	1	LT-1
cuugcauaucucgggCgcuuc	2	1	JCK-2
Uuugcauaucucgggagcuuc	1	1	JCK-2
cuugcauaucucgggagcuuA	1	1	JCK-1
cuugcauaucucgggaAcuuc	1	1	JT-1
cuugcCuaucucgggagcuuc	4	1	JCK-1
cuugcauaucucgggagcCuc	1	1	JCK-2
Uuugcauaucucgggagcuuc	2	1	LT-1
cuugcaCaucucgggagcuuc	4	1	JCK-3

Star Mature

cgagagagagagagagagagagagagaucuaaaaauuaggguuagaagugagcuccuugaaguccaauagaagaucgugcugguagauugagcugcugagcuauggaucccacaagcccuaucucucaaacaauaaaauaagagagagguuagguuuuguuuauaaagagauagauuuguggcuugcauaucucgggagcuucauuuaccuauagaguuagaucuuuguuuggauugaagggagcucuac			
cuugcauaucucgggagcuCc	1	1	LCK-3
cNugcauaucucgggagcuuc	3	1	JCK-2
cuugcauCucucgggagcuuc	2	1	JCK-3
cuugcauaucucgggagcuucU	11	1	LT-3
cuugcauaucucgggagcuucU	21	1	JCK-2
cuugcauaucucgggagcuucU	27	1	JCK-3
cuugcauaucucgggagcuucC	1	1	JCK-3
cuugcauaucucgggagcuucC	2	1	LT-3
cuugcauaucucgggagcuucU	4	1	LT-1
cuugcauaucucgggagcuucC	1	1	JCK-2
cuugcauaucucgggagcuuUa	3	1	JCK-1
cuugcauaucucgggagcuucU	18	1	LCK-3
cuugcauaucucgggagcuucC	1	1	JCK-1
cuugcauaucucgggagcuucU	22	1	JCK-1
cuugcauaucucgggagcuuUa	1	1	JCK-2
cuugcauaucucgggagcuucU	10	1	LT-2
cuugcauaucucgggagcuucG	1	1	LT-1
cuugcauaucucgggagcuucCu	3	1	LT-2
cuugcauaucucgggagcuucUu	1	1	JCK-2
cuugcauaucucgggagcuucUu	1	1	LT-3
cuugcauaucucgggagcuucCu	1	1	LT-1
cuugcauaucucgggagcuucUuu	3	1	LT-1
cuugcauaucucgggagcuuAauuu	1	1	JCK-1
cuugcauaucucgggagcuucauuua	1	0	JCK-3
cuugcauaucucgggagcuucauuua	1	0	JCK-2
cuugcCuaucucgggagcuucauuua	1	1	JCK-2
uuNcauaucucgggagcu	1	1	LCK-3
uugcauaucucgggagcuA	24	1	LCK-2
uugcauaucucgggagcuA	18	1	LCK-1
uugcauaucuGgggagcuuc	1	1	LT-2
uugcauaucucgggagcuuU	4	1	LT-1
uugcauaCcucgggagcuuc	1	1	LT-1
uugcauaucuAgggagcuuc	1	1	LCK-3
uugcauaucucgggagcuuA	1	1	LT-2
uugcCuaucucgggagcuuc	1	1	JCK-2
Cugcauaucucgggagcuuc	1	1	LT-3
uugcauaucucgggagcuuU	3	1	JCK-3
uugcCuaucucgggagcuuc	1	1	LT-2
uugcauaucucgggagcuuU	2	1	LT-3
uugcauaucucgggagcGuc	1	1	JCK-1
uugcauaucuGgggagcuuc	1	1	JCK-1
uugcauaucucgggagcuuU	2	1	JCK-2
uugcCuaucucgggagcuuc	1	1	LCK-3
uugcauCucucgggagcuuc	1	1	JT-1
uugcCuaucucgggagcuuc	4	1	LT-3
uugcauCucucgggagcuuc	1	1	LT-2
uugUauaucucgggagcuuc	1	1	LCK-3
uugcCuaucucgggagcuuc	1	1	LT-1
uugcauaucucgggagcuuA	1	1	LT-1
uugcauaucucgggCgcuuc	1	1	LT-3
uugcauaucucgggagcuuU	6	1	LCK-3
uugcauCucucgggagcuuc	1	1	LT-1
uugcauaGcucgggagcuuc	1	1	JCK-3
uugcauaucucgggagcuuU	6	1	LT-2
uuAcauaucucgggagcuuc	1	1	JCK-2
uuNcauaucucgggagcuuc	1	1	LT-2
uugcaCaucucgggagcuuc	1	1	LT-3
uugcauaucucgggagcuuA	1	1	LT-3
uugcaCaucucgggagcuuca	1	1	LT-2
uugcauaucucgggagcuuUa	1	1	LT-1
uugcauaucucgggagcuuUa	1	1	JCK-1
uugcauaucucgggagcuuAa	1	1	LT-3
uugcauaucucgggagcuucU	2	1	LT-1
uugcauaucucgggagcuucU	1	1	JCK-1
uugcauaucucgggagcuucUu	1	1	JCK-2
uugcauaucucgggagcuucUu	1	1	JCK-1
uugcauaucucgggagcuuUauu	2	1	LT-1
uugcauaucucgggagcuucauC	1	1	LT-3
uugcauaucucgggagcuucauCu	3	1	LT-2
uugcauaucucgggagcuucauuG	1	1	LT-1

Star **Mature**

cgagagagagagagagagagagagagaucuaaaaauuaggguuagaagug**gagcuccuugaaguccaauagaa**gaucgugcuggguagauugagcugcugagcuauggaucccacaagcccuaucucucaaacaauaaaaauaagagagagguuagguuuuguuuauaaagagauagauuuguggcuugcauaucucgggagcuucauuuaccuauagaguuagaucuuug**uuuggauugaagggagcucua**c

uugcauaucucgggagcuuUauuu	2	1	LT-3
uugcauaucucgggagcuucauuu	1	0	JCK-2
uugcauaucucgggagcuucauCu	4	1	LCK-3
uugcauaucucgggagcuuUauuu	4	1	LCK-3
uugcauaucucgggagcuuUauuu	1	1	JCK-2
uugcauaucucgggagcuucauuu	1	0	JCK-1
ugcauaucucgggagcuuU	3	1	LT-3
ugcauaucucgggagcuuU	2	1	LT-1
ugcauaucucgggagcuuU	1	1	LT-2
ugcauaucucgggagcuuU	5	1	LCK-3
ugcauaucucgggagcuucU	12	1	LCK-3
ugcCuaucucgggagcuuca	1	1	LCK-3
ugcauaucucgggagcuucUuu	1	1	LT-1
ugcauaucucgggagcuucauA	5	1	LCK-3
ugcauaucucgggagcuucauuu	1	0	JCK-1
ugcauaucucgggagcuucauuu	2	0	JCK-2
gcauaucucgggagcuuU	1	1	LT-2
cauaucucgggagcuucUuuu	1	1	JCK-3
auaucucgggagcuucauCu	8	1	LCK-3
gagcuucauCuaccuauagagu	1	1	LT-1
agcuucauuuaccuauagaguuaga	1	0	JCK-1
agcuucauuuaccuauagaguuaga	3	0	JCK-2
cauuuaccuauagaguuagaucuuug	2	0	JCK-3
auuuaccuauagaguuagaucuuu	1	0	LT-2
auuuaccuauagaguuagaucuuug	28	0	JCK-3
auuuaccuauagaguuagaucuuug	5	0	LT-1
auuuaccuauagaguuagaucuuug	12	0	LT-2
auuuCccuauagaguuagaucuuug	2	1	LT-2
auuuaccuauagaguuagaucuuug	4	0	LT-3
auuuaccuauagaguuagaucuuug	14	0	LCK-3
auuuaccuauagaguuagaucuuug	7	0	JT-1
auuuaccuauagaguuagaucuuug	20	0	JCK-2
auuuaccuauagaguuagaucuuug	4	0	JT-3
auuuaccuauagaguuagaucuuug	21	0	JCK-1
auuuaccuCuagaguuagaucuuug	1	1	JCK-1
auuuaccuauagaguuagaucuuug	1	0	JT-2
auCuaccuauagaguuagaucuuug	1	1	JCK-2
uCuaccuauagaguuagaucuuug	1	1	LT-1
ucuuuguuuggauugaCggg	1	1	LCK-2
uuuguuuggauugaagggagU	1	1	LT-2
uuuguuuggauugaCgggagcucu	1	1	LT-3
uuuNgauugaagggagcu	1	1	JCK-1
uNuggauugaagggagcu	1	1	JCK-3
uNuggauugaagggagcu	4	1	JCK-1
uuNggauugaagggagcu	1	1	LT-2
uNuggauugaagggagcu	1	1	LCK-1
uNuggauugaagggagcu	1	1	LT-3
uuuggauugaaggNagcuc	1	1	LCK-1
uNuggauugaagggagcuc	2	1	JCK-2
uuuNgauugaagggagcucu	12	1	JCK-1
uuuggauugaaggNagcucu	1	1	JT-2
uNuggauugaagggagcucu	12	1	JCK-3
uNuggauugaagggagcucu	21	1	JCK-2
uuNggauugaagggagcucu	1	1	LCK-1
uuNggauugaagggagcucu	18	1	LT-2
uNuggauugaagggagcucu	20	1	JCK-1
uuuggauugaagggNgcucu	5	1	LCK-1
uuuggauugaaNggagcucu	1	1	JT-2
uuuNgauugaagggagcucu	1	1	JCK-2
uuuggauugaagggagcNcu	2	1	JCK-3
uNuggauugaagggagcucu	2	1	JT-1
uuuggauugaaggNagcucu	2	1	LCK-1
uuuggauugaagNgagcucu	1	1	LCK-1
uuuggauugaaNggagcucu	23	1	JCK-1
uuuggauugaagggaNcucu	1	1	JCK-3
uNuggauugaagggagcucu	1	1	LT-3
uuuggauugaagggagcucN	15	1	JCK-1
uuuNgauugaagggagcucu	1	1	JCK-3
uuuggNuugaagggagcucu	2	1	LCK-1
uuuggauugaaNggagcucu	1	1	JT-1

```
                                                                                                                                                                                                                                        Star                                                                                                                                                          Mature
cgagagagagagagagagagagagaucuaaaaauuaggguuagaaguggagcuccuugaaguccaauagaagaucgugcuggguagauugagcugcugagcuauggaucccacaagcccuaucucucaaacaauaaaauaagagagagguuagguuuuguuuauaaagagauagauuuguggcuugcauaucucgggagcuucauuuaccuauagaguuagaucuuuguuuggauugaagggagcucuac
....................................................................................................................................................................................................................................uuuggauugaagggagcuNu..   1   1   LCK-3
....................................................................................................................................................................................................................................uuuggauugaagggagcuNu..   3   1   JCK-1
....................................................................................................................................................................................................................................uuuggauugaagggagcucuN.   11  1   JCK-1
....................................................................................................................................................................................................................................uuNggauugaagggagcucua.   7   1   LT-2
....................................................................................................................................................................................................................................uuuggNuugaagggagcucua.   1   1   JCK-3
....................................................................................................................................................................................................................................uuuggauugaagggagcuNua.   4   1   JCK-1
....................................................................................................................................................................................................................................uuuggauuNaagggagcucua.   2   1   JCK-3
....................................................................................................................................................................................................................................uuuggauugaagggagcucNa.   36  1   JCK-1
....................................................................................................................................................................................................................................uuuNgauugaagggagcucua.   4   1   JCK-3
....................................................................................................................................................................................................................................uuuggauugaagggagcNcua.   1   1   JCK-3
....................................................................................................................................................................................................................................uuuggauugaaNggagcucua.   26  1   JCK-1
....................................................................................................................................................................................................................................uuuggaNugaagggagcucua.   1   1   JCK-3
....................................................................................................................................................................................................................................uuNggauugaagggagcucua.   2   1   LT-3
....................................................................................................................................................................................................................................uNuggauugaagggagcucua.   38  1   JCK-2
....................................................................................................................................................................................................................................uNuggauugaagggagcucua.   33  1   JCK-3
....................................................................................................................................................................................................................................uNuggauugaagggagcucua.   37  1   JCK-1
....................................................................................................................................................................................................................................uuuNgauugaagggagcucua.   19  1   JCK-1
....................................................................................................................................................................................................................................uuuggauugaagggaNcucua.   1   1   JCK-3
....................................................................................................................................................................................................................................uNuggauugaagggagcucua.   1   1   LT-1
....................................................................................................................................................................................................................................uuuggauugaagggagcucuNc   1   1   JT-2
.....................................................................................................................................................................................................................................uNggauugaagggagcucu..   1   1   JCK-2
.....................................................................................................................................................................................................................................uNggauugaagggagcucu..   1   1   JCK-1
.....................................................................................................................................................................................................................................uuggauugaagggagcucuN.   1   1   JCK-1
.....................................................................................................................................................................................................................................uNggauugaagggagcucua.   3   1   JCK-2
.....................................................................................................................................................................................................................................uuggauugaagNgagcucua.   2   1   JCK-1
.....................................................................................................................................................................................................................................uNggauugaagggagcucua.   2   1   JCK-1
.....................................................................................................................................................................................................................................uugNauugaagggagcucua.   1   1   JCK-1
.....................................................................................................................................................................................................................................uuggauugaagggaNcucuac   1   1   LCK-1
```
